# Human Breast Milk Provides Better Antioxidant Capacity than Infant Formula

**Published:** 2010

**Authors:** Mohammad Reza Oveisi, Naficeh Sadeghi, Behrooz Jannat, Mannan Hajimahmoodi, Abd-ol-Azim Behfar, Forouzandeh Jannat, Fariba MokhtariNasab

**Affiliations:** a *Department of Drug and Food Control, School of Pharmacy, Tehran University of Medical Sciences, Tehran, Iran.*; b * Food and Drug Laboratory Research Center, Ministry of Health and Medical Education, Tehran, Iran.*; c * Food Sciences and Medical Hydrology Department, School of Pharmacy, Ahvaz Jundishapur University of Medical Sciences, Ahvaz,Iran.*; d * Arash Hospital, Tehran University of Medical Sciences, Tehran, Iran.*

**Keywords:** Total antioxidant capacity, Human breast milk, nfant formula, FRAP

## Abstract

Human milk contains all of the constituents that are required for the optimal growth and development of a neonate. It supports the development of brain, immune, and physiological systems. This study aimed to consider the significance of breast milk in preventing oxidative stress by comparing total antioxidant capacity (TAC) in breast and formula milk for premature infants, demonstrating the relationship between TAC in breast milk and postnatal age in days.

The Ferric reducing antioxidant power assay (FRAP) method was used to spectophotometrically measure of TAC in breast and formula milk. One hundred and fourty (n = 140) lactating mothers agreed to participate in the study. TAC was also measured in two brands of formula milk (n = 80).

The Range of TAC in human breast milk was 234.27-1442.31 μM and in two formula was 160.04-630.92 μM. The average TAC was significantly higher in breast milk (642.94 ± 241.23 μM) compared to formula milk (280.986 ± 100.34 μM) p < 0.0001. The TAC of breast milk was increased with some nutritional parameter such as increased consumption of cheese, vegetables, fruits, bread and nuts. Infants’ height at the birthday was directly correlated with antioxidant capacity of breast milk, whilst a reversed correlation was observed between TAC in breast milk and infant age.

Based on our results, it is concluded that the TAC of breast milk is varied and affected by nutrition. It is alo observed that TAC is significantly higher in breast milk than formula, which means that breast milk provides better antioxidant potency than infant formula.

## Introduction

Oxidative stress tends to occur during the neonatal period because the younger gestational age makes the lower antioxidant capacity (AC) and a lower AC tends to result in an increase in free radicals (FR) and reactive oxygen species (ROS) (1). Cell damage due to oxidative stress during the neonatal period has been associated with disease (2) for example, oxidative stress has been reported in association with necrotizing entero- colitis if the site of the damage is in the digestive tract (3), with chronic lung disease if in the lungs (4), retinopathy of prematurity if in the eyes (5), and periventricular leukomalacia and intraventricular hemorrhage if in the brain (6, 7).

Determining how to avoid oxidative stress is an important factor for improving prognosis. Broadly, there are two ways to prevent oxidative stress. One is to avoid factors that trigger the production FR and ROS. The other is to strengthen AC, which protects the body from cell damage by scavenging FR and ROS when they increase. Breast milk is mostly the only food for many infants under 6 months (8-11). Antioxidants of the human milk still have not been completely defined but the information taken is the face: tocopherol, cysteine, carotenoids, glutathione peroxidase, catalase, superoxid dismutase, coenzyme Q, vitamin A, vitamin E, vitamin C, etc.

Indeed, breast milk is the best wish prepared formula for infant, rather than industrial prepared formula milk. It is also different of looks in micro and macro elements, although based on short- term studies; it is known that human breast milk has greater AC than formula (12-13).

Breast feeding has been reported to be clinically efficient and significant when compared with formula feeding. For example, compared to formula feeding cohorts, breast feeding showed a 1 ⁄ 10 decrease in risk of necrotizing enterocolitis development (14), better neurodevelopment (15) and a decrease in the retinopathy of prematurity (16). As there have been no reports on Iranian mothers breast milk total antioxidant capacity (TAC), the present study was conducted to elucidate the significance of breast feeding with a view toward antioxidant capacity, comparing to infant formula and to clarify the relationship between AC in breast milk and the postnatal age characteristics.

## Experimental

All solvents and chemicals were of analytical grade and obtained from Merck company (Darmstadt, Germany). Double – distilled deionized water was used for the preparation of aqueous solutions.

*Sample preparation*

A number of one hundred and fourty lactating mothers (n = 140), of whom four had delivered premature infants (< 2450 g, ≤ 37 weeks gestational age) and the remaining had full-term infants, agreed to participate in the study. The questionnaire included demographic variables: self reported age, history of diseases, nutritional status and postnatal age characteristics. The questionnaire was performed by a trained interviewer. The project was approved by Ethics Committee of Tehran University of Medical Sciences. A total of 140 human breast milk samples were collected from 4 clinics in Tehran, Iran. Samples were immediately frozen and stored at – 20 ºC until analysis. Also a number of 80 infant formulas were purchased from drug stores.

*Measurement of total antioxidant activity*

The FRAP (Ferric Reducing Antioxidant Power assay) procedure described by Benzie and strain was followed (17). The method is based on the reduction of a ferric–tripyridyltriazine complex to its ferrous colored form in the presence of antioxidants. Briefly, the FRAP reagent contained 5 mL of a (10 mM) TPTZ (2,4,6,-tripyridyl -s-triazine) solution in 40 m MHCl plus 5 mL of FeCl3 (20 mM) and 25 mL of acetate buffer and was prepared freshly warmed at 37 ºC.

Before analysis, the breast milk samples were thawed at 25 ºC and shaked for 5 min (200⁄min), 50 μL of each sample of breast milk or infant formula (30 mL of infant formula was performed) was transferred to two test tubes and mixed with 1.5 mL of FRAP reagent and 1.5 mL of deionized distilled water. The absorbance of the two mixtures was measured spectrophotometrically (593 nm) against FRAP solution as blank, after incubation at 37°C for 10 min. The difference between these two absorbences indicated the TAC content of the samples. A series of concentration of FeSO4 including 1000, 750, 500, 250 and 125 μM were used for construction of calibration curve and the absorbences of these solution were measured as described for sample solutions.

## Results and Discussion

TAC was detected in 140 samples of human breast milk at mean concentration of 642.94 ± 241.23 μM, ranging between 234.27-1442.31 μM (Table 1). The Range of TAC in formula was160.04-630.92 μM. 

**Table 1:** Antioxidant content of breast milk and infant formula

	N	Mean ± SD (μM)
Human milk	140	642.94 ± 241.23
Formula milk	80	280.86 ± 100.34

The obtained data indicated that TAC is clearly higher in breast milk than formula (p < 0.0001). As shown in Table 2, the results showed that the TAC in breast milk will increase with increase in consumption of cheese, vegetables, fruit, bread and nuts. (Table 2, p < 0.05).

**Table 2:** The human breast milk TAC in relationship to the amount of dairy consumption and bread, nuts, fruit, and vegetables

**Mother’s food intake **	**TAC of breast milk mean ± SD (**μM**) **
Less than 30 g cheese	596.37 ± 221.48
30 g cheese	648.66 ± 261.64
More than 30 g cheese	6769.75 ± 196.75
Less than 30 g of bread a day	564.62 ± 206.17
30 g of bread a day	731.67 ± 259.82
30 g to 60 g of bread a day	552.75 ± 144.81
More than 60 g bread a day	585.71 ± 176.81
No nut consumption	696.49 ± 270.91
one glass nut for week	617.93 ± 256.92
More than one glass nut for week	547.91 ± 176.05
No vegetable consumption a day	685.23 ± 270.38
One Meal vegetable a day	660.69 ± 201.88
More than one meal vegetables a day	441.94 ± 201.62
1 to 2 fruits a day	617.82 ± 171.57
3 to 5 fruits a day	633.93 ± 272.40
More than 5 fruits a day	794.61 ± 207.66

This is due to the high antioxidant contents of dairy products (which provide tocopherols or vitamin E, vitamin A, carotenoids, some fat soluble micronutrients) (18, 19), fruits and vegetables (which contain vitamin C, beta-carotene, anthocyanins, sterols, lignans) (20, 21), cereals (containing phytostrols, lignans, Zn, vitamin E) (22) and nuts (which contain saponins, tannins, coenzyme Q, flavonoids, vitamin E, Se, vitamin A, etc) (23). 

Infant height at the birthday was directly correlated with antioxidant capacity of breast milk (Table 3) and it may be suggested as a consequence of nutritional and food intake status in pregnancy period. Also, our results showed that the TAC of breast milk will decrease as the infant grows. 

**Table 3:** Correlation between the TAC of breast milk and growth and anthropometric measurements of infant

	**p-value **	**Pearson correlations **
Infant age (month)	0.002	-0.270
Height at birth (cm)	0.010	0.237
Weight at birth (kg)	0.940	-0.007
Head circumference at birth (cm)	0.251	0.157

In a similar work, Ezaki *et al. *studied the TAC content of mothernal milk by FRAP assay and they found the same results and reported that TAC in breast milk (n = 56, 3807 ± 103.5 μM) is higher than in formula (n = 12, 2671 ± 96 μM) (p < 0.0001). The study performed on 56 samples of breast milk collected from mothers of premature infants born with a mean ± SD gestational age of33 ± 4 weeks that was collected at postnatal age 39 ± 43 days in Japan (24). 

Also, in another study, Aycicek*et al*. reported that breast milk provides better antioxidant power than formula. Fifty-four healthy term infants 3 to 6 month of age were fed breast milk or a cow’s milk modified formula. The report showed that no significant differences were observed between groups with respect to growth or anthropometric measurements. Plasma TAC and vitamin C levels were significantly higher in the breast-fed group than in the formula-fed group. Plasma total peroxide levels and the oxidative stress index which are biomarkers of oxidative status were higher in the formula-fed group than those in the breast-fed group (p < 0.05) (25) and in a study the concentration of lead; as a toxic element was significantly more than standard and labeled value (26). 

Quills studied thirty healthy breastfeeding women provided colostrums, transition – milk and mature – milk samples. The report showed that coenzyme Q is present in breast milk with high concentration in mothers of full – term infants. Coenzyme Q in breast milk decreases through lactation in mothers delivering full – term infants. Also coenzyme Q , alpha- and gamma tocopherol concentrations in human milk directly correlates with the antioxidant capacity of the milk (27). These data are in agreement with our results about the TAC of breast milk which will decrease with time after birthday. 

Shoji studied suppressive effects of breast milk on oxidative DNA damage in very low birth weight infants. The study indicated that in the breast fed group, urinary 8- Hydroxy deoxy guanosine excretion, which is known to be a non-invasive marker for in vivo oxidative DNA damage, was significantly lower than that in the formula fed group. The study preformed on a breast fed group of 15 infants and a formula fed group of 14 infants in Nagaoka Hospital in Japan (14). 

Superoxide dismutase and glutathione peroxidase (antioxidant enzymes) content of human milk from mothers of premature and full – term infants during the first 3 months of lactation were studied by L’Abbe *et al*. Nine samples were collected from each of 15 mothers of full-term infants and 19 mothers of healthy pre-term infants. The report showed that in both groups, total mill units of glutathione peroxidase and Se-glutathione peroxidase per milligram protein and superoxide dismutase per milligram protein increased. Se-glutathione peroxidase was high in the pre-term group at week 1, but the superoxide dismutase activity was higher throughout the entire study in the full-term milk (28). 

In this study, it was was shown that the total antioxidant capacity than formula, and revealed that breast milk provides better antioxidant power than does infant formula. Thus, along with other benefits, human breast milk is preferred to be used as a health protective antioxidant food for young children.
